# Sub-axillary cosmetic incision versus single-incision thoracoscopic surgery for primary spontaneous pneumothorax

**DOI:** 10.1186/s13019-023-02319-w

**Published:** 2023-07-12

**Authors:** Yuan-Liang Zheng, Ri-Sheng Huang

**Affiliations:** Department of Thoracic Surgery, Wenzhou Central Hospital, The Dingli Clinical College of Wenzhou Medical University, the Second Affiliated Hospital of Shanghai University, Wenzhou, Zhejiang China

**Keywords:** Pneumothorax, Single-incision thoracoscopic surgery, Sub-axillary cosmetic incision, Propensity score matching, Video-assisted thoracoscopic surgery

## Abstract

**Background:**

In recent years, single-incision thoracoscopic surgery (SITS) has been increasingly applied as an optimal treatment option for primary spontaneous pneumothorax (PSP). However, most SITS techniques are used in the fourth to sixth intercostal space between the anterior axillary and mid axillary lines. To find out more concealed incisions, this study performed PSP surgery via the sub-axillary cosmetic incision (SACI) technique.

**Methods:**

A total of 128 PSP patients were subjected to video-assisted thoracoscopic surgery (VATS) between January 2017 and January 2019 at our institution. These patients were evaluated and assigned into SACI (n = 21) and SITS (n = 57) groups. Propensity score matching (PSM) was performed based on patients’ backgrounds, and the enrolled cohort was divided into 21 pairs. The incision satisfaction was assessed at 2 weeks and 6 months post-surgery.

**Results:**

The 21 pairs with matching baseline characteristics in the two groups did not exhibit significant differences in their backgrounds and surgical results. However, compared with the SITS group, the operation time was longer in the SACI group (*p* = 0.013). There were no post-operative complications in both groups. At 2 weeks and 6 months, incision satisfaction scores in the SACI group were significantly lower than those in the SITS group (p = 0.022 and p = 0.039, respectively). There were no recurrences of ipsilateral pneumothorax in both groups.

**Conclusions:**

SACI is a safe and feasible surgical method for PSP treatment. In addition, incision concealment can be used for patients with incision needs.

## Background

Among adolescents. PSP is the most common chest disease [1]. The standard surgical procedure for these patients is VATS bullectomy. Compared with traditional thoracotomy, conventional three-port VATS provides significant benefits in reduced pain and respiratory function recovery [2]. Gonzalez et al. [3] reported that compared to the conventional multiport procedures, SITS has better cosmetic effects and less incision pain. However, most SITS are made in the skin at the fourth to sixth intercostal space between the anterior axillary and mid axillary lines [4, 5]. Further, Hee et al. [6] reported 70.4% satisfaction from wound scarring. Therefore, there is a need to create a more concealed incision that can improve wound scarring comfort in PSP patients. This study aimed at assessing the safety and the effectiveness of the SACI technique, when compared to SITS, for treating PSP patients.

## Methods

Between January 2017 and January 2019, we retrospectively reviewed the surgical records of patients who underwent VATS for PSP. Written and oral informed consent was received by all patients. The present study was approved by the ethics committee of Wenzhou Central hospital (L2017-02-140).

The inclusion criteria for this study were: [1] patients with primary spontaneous pneumothorax; [2] patients whose location and extent of bullae was verified by the pre-operative high-resolution computed tomography scans of the chest; [3] patients aged between 15 and 40 years; [4] patients without insertion of a chest tube before surgery.

To reduce deviations between the groups, the exclusion criteria were: [1] patients with no history of lung diseases, such as pulmonary fibrosis, pulmonary tuberculosis, or chronic obstructive pulmonary disease; [2] patients with no history of ipsilateral lung surgery; [3] patients with co-morbidity of hemopneumothorax; [4] patients who were unwilling to accept surgical treatment; [5] patients who did not agree to sign the informed consent; [6] patients with life-threatening tension pneumothorax where emergency thoracic closed drainage was required and [7] patients with incomplete records.

The collected demographic data included age, sex, height, weight, intra-operative adhesion, smoking history, the involved side, operation time, blood loss during surgery, post-operative drainage, duration of post-operative hospital stay, post-operative complications and post-operative pain scores, which were evaluated using visual analog scales from 0 (no pain) to 10 (worst pain ever experienced). Pain scores were recorded at 24 h, 48 h 72 h and on the first week after surgery. All patients were followed up via post-operative telephone interviews for at least 6 months. Postoperative recurrence during follow-up period was defined as ipsilateral recurrence of pneumothorax requiring intervention treatment. The follow-up was conducted to evaluate the level of satisfaction with the surgical wound and recorded in 4 grades at 2 weeks and 6 months after surgery. Detailed guidelines for the scoring scale are shown in Table 1.

**Table 1:** Scoring scale

Grades	Scores	Incision performance
Excellent	1	Good concealment of incision and scarless hyperplasia
	2	Good concealment of incision and mild scar hyperplasia
Good	3	General concealment and mild scar hyperplasia
	4	General concealment and moderate scar hyperplasia
Fair	5	General concealment and severe scar hyperplasia
	6	Poor concealment and mild scar hyperplasia
Poor	7	Poor concealment and moderate scar hyperplasia
	8	Poor concealment and severe scar hyperplasia

### Surgical technique

Using a double-lumen endotracheal tube and one-lung ventilation, two groups of surgeries were performed on the patients under general anesthesia. First, the patients were placed in a lateral decubitus position. Our surgical principle for PSP was to perform lung wedge resection with the same types of endoscopic stapler. No residual lesions were identified intra-operatively. Further, a water inundation test was performed to check for air leakage. Finally, a 20 F chest tube was inserted into the top of the pleural cavity and connected to a water-sealed bottle through the incision site. An intradermal suture was performed around the tube with 3 − 0 Vicryl, and the chest tube was fixed with a suture knot that was not tied under the skin. The thread was removed after the chest tube was removed, and the 3 − 0 Vicryl suture was tightened further.

### SITS technique

**Fig. 1:**
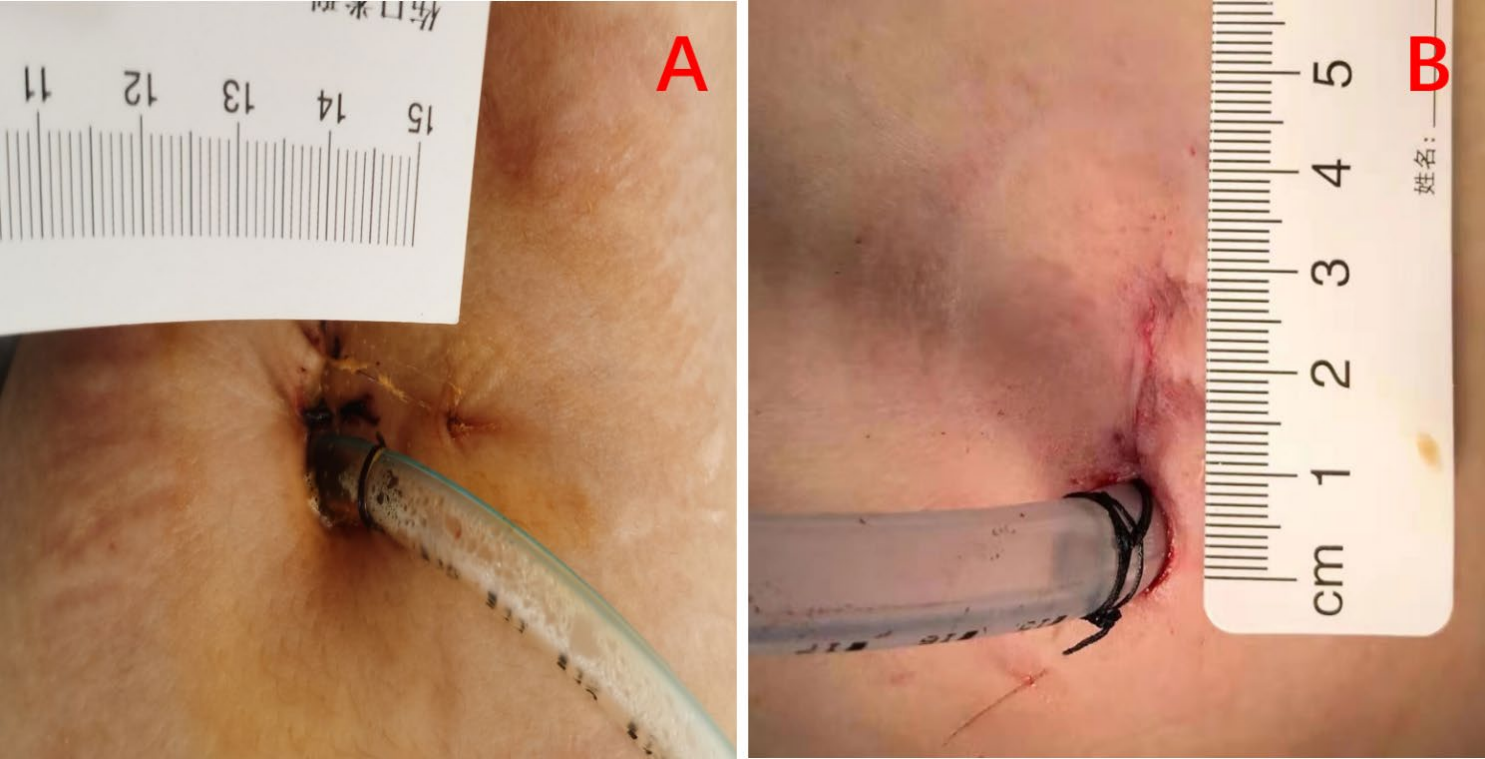
**A** represent a SACI technique with a 2.5-cm incision to place a chest drainage tube, **B** represent a SITS technique with a 2.5-cm incision to place a chest drainage tube

A 2.5-cm long skin incision was made in the fourth or fifth intercostal space between the anterior axillary line and mid axillary line. The protective sheath was placed in the incision. The 5-mm, 30^0^ thoracoscopy was introduced through the incision, to keep the thoracoscopy close to the mid axillary line. The whole chest was explored with lung collapse, whether there were adhesions, the location and the size of the bulla of the lung. Finally, the bulla was removed with the same type of endoscopic stapler (Fig. 1).

### SACI technique

A 2.5-cm long skin incision was made in the axillary fold, exposing the intercostal space with a hook (because the skin incision was not parallel to the intercostal space, it was about 90 degrees), selected at the third intercostal space. The protective sheath was placed in the incision, the 5-mm, 30^0^ thoracoscopy was introduced through the incision, to keep the thoracoscopy close to the mid axillary line. The whole chest was explored with lung collapse, whether there were adhesions, the location and the size of the bullae of the lung, and the bullae were removed with the same type (Johnson Echelon 45) of the endoscopic stapler (Fig. 1).

Propensity score matching (PSM).

PSM was performed to reduce the biases in patient selection. The logistic regression was used to calculate the propensity score. The covariates of age, sex, weight, height, side involved, intraoperative adhesions, and smoking history included in the calculation, which might affect the comparison result of the two groups.

### Statistical analysis

Data were analyzed using the Statistical Product and Service Solutions (SPSS) software (version22.0, SPSS Inc., Chicago, IL, USA) program. Categorical variables were presented as percentages and compared by the chi-square test or Fisher’s exact test. Shapiro Wilk was used for the normality test (when p > 0.05, the data is close to normal distribution). Continuous variables with normal distribution were expressed as medians ± standard deviation and compared by student’s t-test. The Mann-Whitney U test was used to compare means of continuous variables with non-normal distribution; continuous variables were summarized as median and interquartile range, with statistical significance set at *p* < 0.05.

## Results

**Fig. 2:**
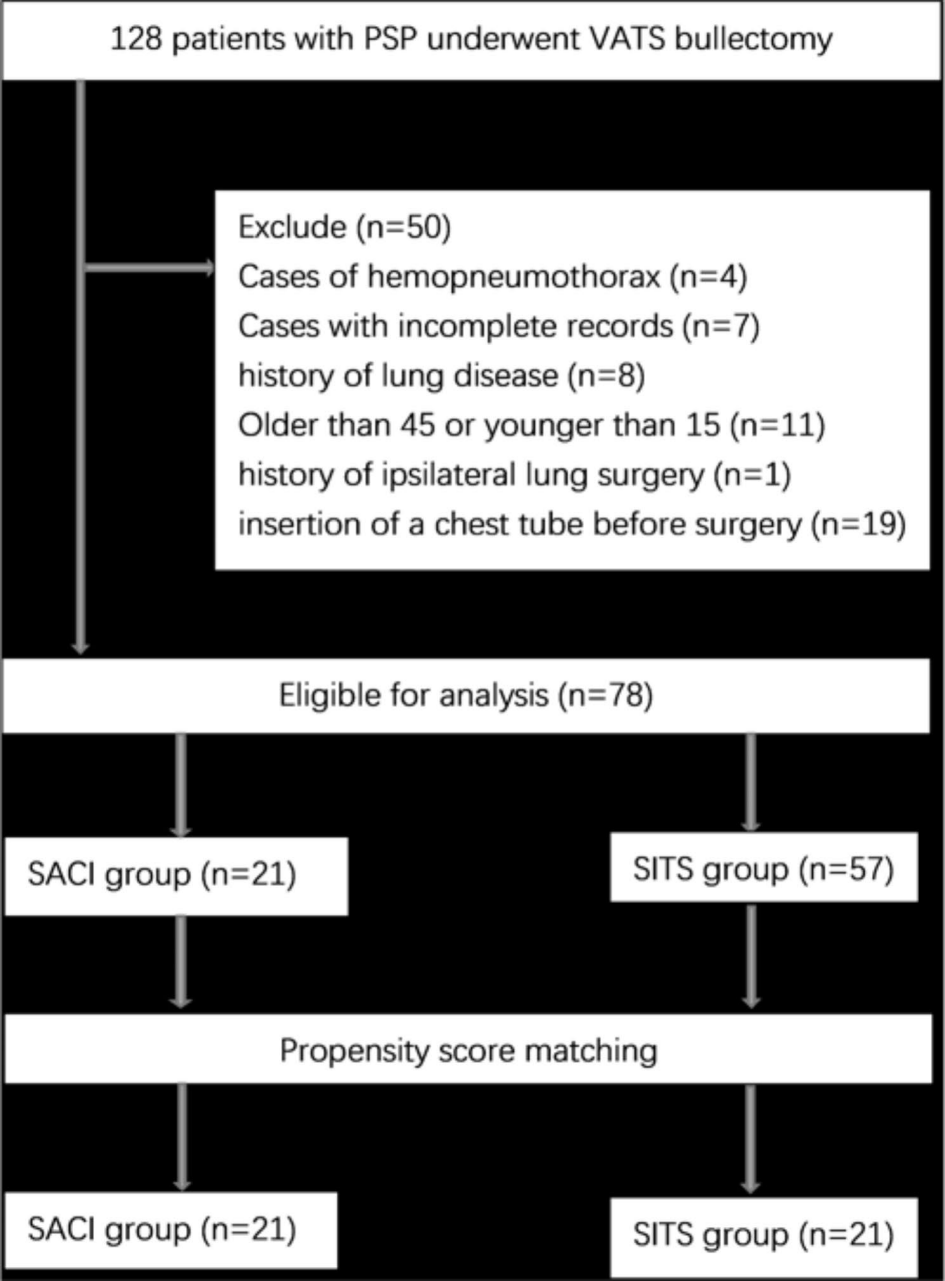
Details of the study enrollment

**Fig. 3:**
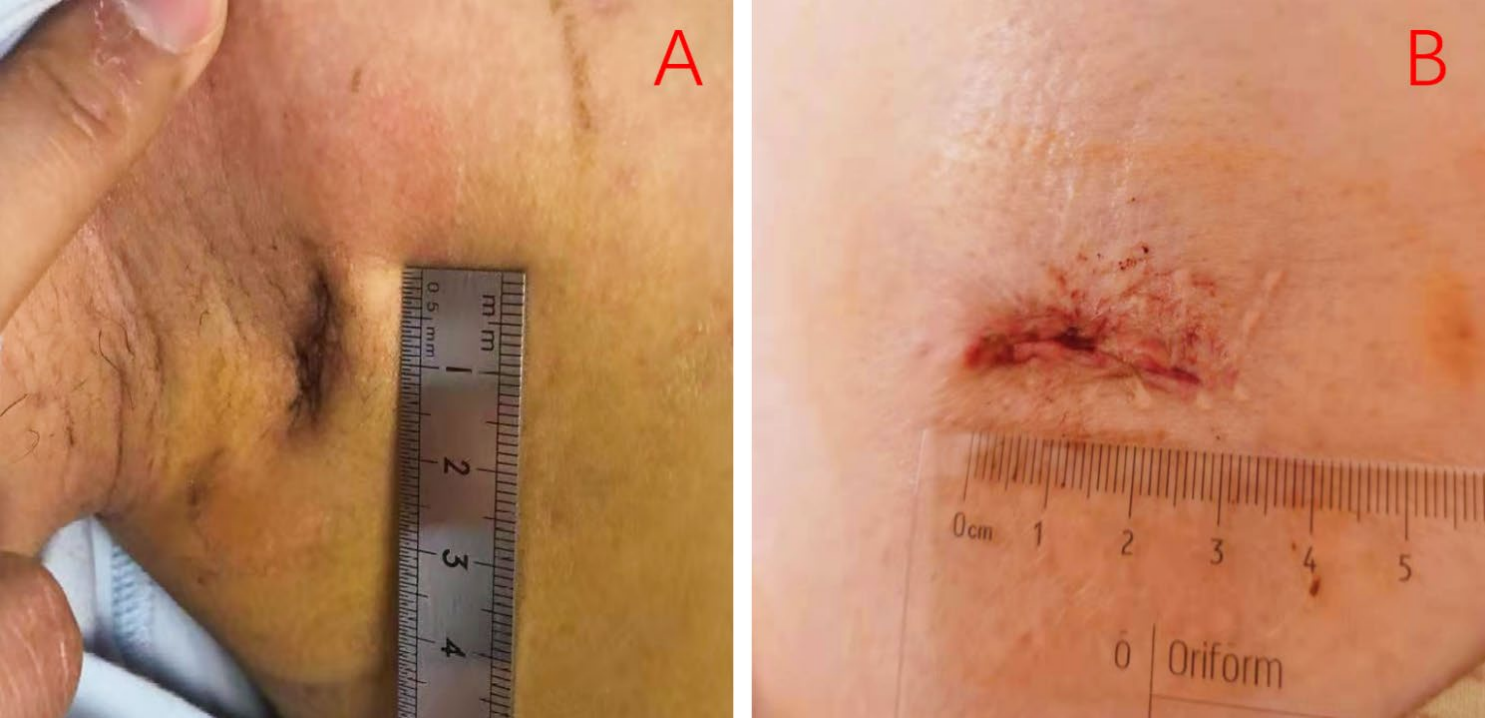
**A** represent the wound healing in two weeks after operation with SACI technique, **B** represent the wound healing in two weeks after operation with SITS technique

**Fig. 4:**
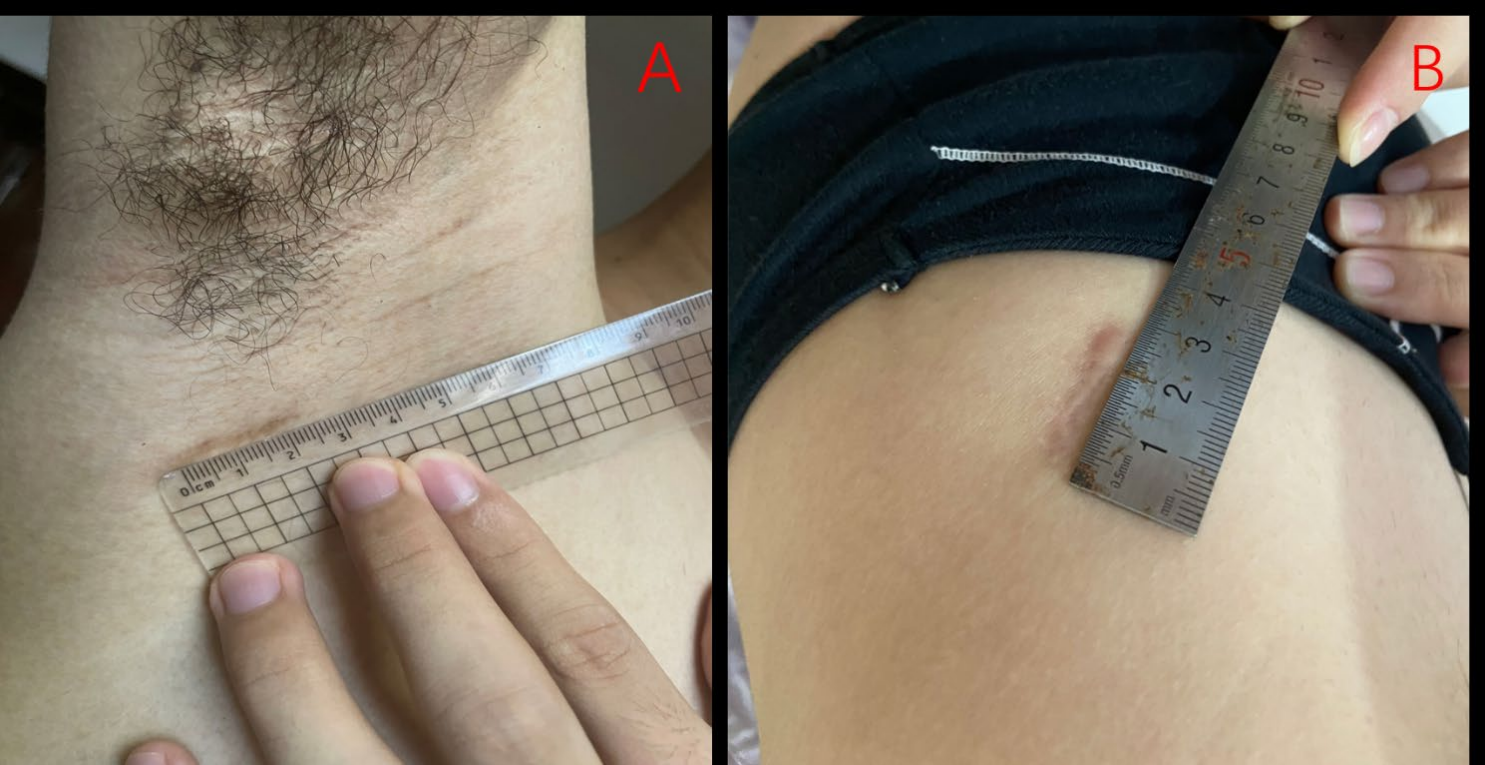
**A** represent the wound healing in 6 months after operation with SACI technique, **B** represent the wound healing in 6 months after operation with SITS technique

A total of 128 consecutive patients were subjected to VATS for PSP treatment. Among them, patients with spontaneous hemopneumothorax (n = 4), incomplete medical records (n = 7), lung disease (n = 8), aged > 40 or < 15 years (n = 11), history of ipsilateral lung surgery (n = 1), and insertion of chest tube before surgery (n = 19) were excluded from the analysis. Seventy-eight patients were finally included in our study. 21 were treated with SACI while 57 were treated with SITS. Baseline characteristics of the 78 patients are shown in Table 2. Patients in both groups were matched 1-to-1 with a caliper distance of 0.2, no replacement was required by SACI group, after PSM, matching resulted in 21 patients in each group. The study selection process was as shown in Fig. 2. Mismatch analysis revealed significant differences in age and height of the two groups (p = 0.025 and p = 0.038, respectively). Before PSM, median age for the SACI group was 24.9 ± 6.6 years, with a median height of 169.8 ± 5.3 cm. Seventeen patients were male (81%), 7 patients smoked (33.3%), while 2 patients had pleural adhesions (9.5%). The median age for the SITS group was 22. 1 ± 3.9 years, while the median height was 172.6 ± 5.2 cm. Out of the 57 patients, 36 were male (63.2%), 13 were smokers (22.8%), while 10 patients had pleural adhesions (17.5%). Finally, 21 pairs of patients were matched for this study. After PSM, there were no significant differences with regards to any of the baseline characteristics. Baseline characteristics post PSM are presented in Table 2. Post PSM, the median ages for the SACI and SITS groups were 24.9 ± 6.6 and 24.0 ± 4.4 years, respectively. Surgical and post-operative outcomes after PSM are shown in Table 3. Patients receiving SACI had significantly longer surgical times than those subjected to SITS (*p* = 0.013). However, both groups exhibited comparable hospital stays after surgery, post-operative complications, pain scores on post-operative days, blood loss during surgery, and post-operative drainage. Compared to the SITS group, the SACI group had markedly low patient satisfaction scores at 2 weeks (Figs. 3) and 6 months (Fig. 4) after surgery (p = 0.022 and p = 0.039, respectively). Notably, during the following-up period, there were no cases of post-operative complicationsin either group.

**Table 2:** The baseline characteristics of patients

Characteristics	Before propensity score matching				After propensity score matching		
	**SACI group (n = 21)**	**SITS group (n = 57)**	***p*** **-value**		**SACI group (n = 21)**	**SITS group (n = 21)**	***p*** **-value**
Age(years)	24.9 ± 6.6	22.1 ± 3.9	0.025^a^		24.9 ± 6.6	24.0 ± 4.4	0.17
Sex			0.135				1.00
Male	17(81%)	36(63.2%)			17(81%)	17(81%)	
Female	4(19%)	21(36.8%)			4(19%)	4(19%)	
Weight(kg)	57.1 ± 5.0	57.5 ± 4.3	0.752		57.1 ± 5.0	56.8 ± 3.9	0.467
Height(cm)	169.8 ± 5.3	172.6 ± 5.2	0.038^a^		169.8 ± 5.3	170.3 ± 5.2	0.449
Side involved			0.993				0.938
Right	13(61.9%)	36(63.2%)			13(61.9%)	14(66.7%)	
Left	6(28.6%)	16(28.1%)			6(28.6%)	5(23.8%)	
Bilateral	2(9.5%)	5(8.8%)			2(9.5%)	2(9.5%)	
Smoking			0.345				0.739
Yes	7(33.3%)	13(22.8%)			7(33.3%)	6(28.6%)	
No	14(66.7%)	44(77.2%)			14(66.7%)	15(71.4%)	
Pleural adhesion			0.605				0.663
Yes	2(9.5%)	10(17.5%)			2(9.5%)	4(19%)	
No	19(90.5%)	47(82.5%)			19(90.5%)	17(81%)	

SACI = sub-axillary cosmetic incision; SITS = single-incision thoracoscopic surgery; ^a^p<0.05, the difference was statistically significant. Values are medians ± standard deviation for continuous variables or cases (%) for categorical variables

**Table 3:** Postoperative events after propensity score matching

Characteristics	SACI group (n = 21)	SITS group (n = 21)	*P*-value
Operation time (min)	71.1 ± 11.6	58 ± 11.4	0.013^a^
Blood loss during surgery (ml)	29.5 ± 18.0	27.1 ± 7.2	0.571
Postoperative drainage (ml)	134.3 ± 40.6	134.8 ± 31.4	0.971
Postoperative hospital stays (d)	2.5 ± 0.7	2.4 ± 0.6	0.629
Postoperative complication			1.00
Yes	0	0	
No	21 (100%)	21 (100%)	
Pain scores after surgery (interquartile)			
24 h median	3(1–9)	4(1–10)	0.878
48 h	2(1–5)	2(1–6)	0.432
72 h	2(1–3)	2 (1–3)	0.776
The first week	1(1–2)	1(1–2)	1.0
Wound satisfaction scores (2-weeks)	3(1–5)	4(2–6)	0.022^a^
Wound satisfaction scores (6-months)	3(1–6)	4(2–9)	0.039 ^a^

SACI = sub-axillary cosmetic incision; SITS = single-incision thoracoscopic surgery; ^a^p<0.05, the difference is statistically significant. Values are medians ± standard deviation or median (interquartile) for continuous variables or cases (%) for categorical variables

## Discussion

Compared with conventional thoracotomy, VATS has many advantages [7]. The conventional three-port VATS bullectomy is the most popular approach for PSP surgery. However, patients who are subjected to conventional three-port VATS usually complain of post-operative pain and paresthesia [8]. To address these post-operative complications, efforts have been made to reduce the length of incisions, as the SITS approach reportedly had better cosmetic effects and low rates of post-operative pain and paresthesia [9]. However, conversion of three holes to a single hole is a long process, requiring technological advances and appropriate instruments. For instance, Racco et al. [10] introduced SITS for treating PSP in 2004, however, the SITS technique has not been widely used in the last decade. Its wide applications were limited by instrument collision that required a relatively long skin incision. In 2014, Ng et al. [11] developed novel, smaller, and more specialized procedure-specific instruments for SITS, which allowed reduction of incision sizes. Since then, due to advances in endoscopic technologies and use of relatively new instruments, single hole thoracoscopic bullectomy has become simple. Moreover, the emergence of new instruments has led to increased adoption of the single-hole thoracoscopic bullectomy by of surgeon. Even though the incision is smaller than before, the wound scarring is not concealed enough, which affects patients’ satisfaction post-surgery. Notably, cosmetic requests are frequent among younger patients. Therefore, we used the SACI technique, which has a more concealed way of suturing and achieved better satisfaction, especially for young patients. Satisfaction scores for the SACI technique were markedly higher than those of the SITS approach.

In this study, we excluded the histories of hemopneumothorax and lung diseases, mainly to reduce the confounding factors. Patients in the SACI group could not retain the chest tubes before operation, while patients with hemopneumothorax often have to retain the chest tube before operation as an intervention measure, and even require emergency surgical interventions. Patients with a history of pulmonary diseases may be complicated with extensive adhesions of chest cavities. Because of visual field limitations in the SACI group, it is not conducive to release adhesion at the diaphragm during operation, which complicated the operation in the SACI group, relative to the SITS group. For patients aged 18–40, young patients have a strong aesthetic desire for incision.

We present 128 cases of VATS for PSP, and the 5-mm, 30^0^ thoracoscopy was used in all bullectomy procedures. This provided enough visual field for surgery, which was especially suitable for the 2.5-cm incision. Currently, 5 mm thoracoscopy is the best choice for reducing the size of the incision without affecting the operation field of vision. There was no conversion to open thoracotomy in both groups. Neither group had post-operative complications, indicating that the operation technology was safe and feasible. The high recurrence probability of pneumothorax after bullectomy can be reduced by using pleural abrasion during the operation [12]. However, Hatz et al. [13] showed that pleural abrasion can increase the post-operative thoracic drainage, delay extubation, lead to post-operative complications and secondary operation difficulty. In this study, none of the surgical patients received any treatment for pleural abrasion and had no recurrence of pneumothorax during follow-up. However, routine use of pleural abrasion is not recommended, especially in young patients.

Differences in hospital stay after surgery, post-operative complications, pain scores on post-operative days, blood loss during surgery, and post-operative drainage between the groups were insignificant. However, the operation time of the SACI group was significantly longer than that of the SITS group (p = 0.013). In addition to the longer time associated with chest adhesion, the main reason for the long operation time was the surgical path. Due to the high position of sub-axillary cosmetic incision, and the relatively limited vision field, it takes more time to remove the bullae in the lower lobe using an endoscopic stapler.

Satisfaction of incision in the SCAI group was higher than the SITS group. First, incision in the SACI group was at the axillary dermatoglyphic. After the patients’ hands had naturally dropped, the incision position could hardly be seen. Given the convenience of post-operative follow-up, we evaluated the incision at 2 weeks postoperatively; however, at this time, the incision was not old enough for scar hyperplasia. Therefore, the incision looked smooth, and the satisfaction score was high. However, the overall satisfaction with incision of patients at 6 months after operation was lower than at 2 weeks post-operation. This could have been because some patients in the SACI group had obvious scar hyperplasia from incision at 6 months after operation. Axillary hairs of these patients did not completely cover the scar, resulting in a decrease in post-operative satisfaction. Since this study had a short-term follow-up of 6 months after operation, it was unclear whether patients’ satisfaction with incision would change at one or more years after operation.

Since the treatment methods for pneumothorax were not randomly assigned, baseline characteristics for the two treatment groups differed. Applications of PSM reduced the deviation characteristics caused by unbalanced covariates. This study had some limitations. Although we used PSM to reduce the selection bias, the total number of patients was small, and the post-operative follow-up time was short.

## Conclusions

In summary, SACI is a safe and effective technique for PSP treatment of selected cases, especially for patients with high requirements of wound concealment.

## Data Availability

The raw data supporting the conclusions of this article will be made available by the authors, without undue reservation. Further inquiries can be directed to the corresponding author.
